# Use of a Web 2.0 Portal to Improve Education and Communication in Young Patients With Families: Randomized Controlled Trial

**DOI:** 10.2196/jmir.2425

**Published:** 2013-08-23

**Authors:** Lena Hanberger, Johnny Ludvigsson, Sam Nordfeldt

**Affiliations:** ^1^Division of PediatricsDepartment of Clinical and Experimental MedicineLinköping UniversityLinköpingSweden; ^2^Division of Child and Adolescent PsychiatryDepartment of Clinical and Experimental MedicineLinköping UniversityLinköpingSweden

**Keywords:** type 1 diabetes, children, adolescent, health information technology, patient education, intervention studies, HbA1c

## Abstract

**Background:**

Diabetes requires extensive self-care and comprehensive knowledge, making patient education central to diabetes self-management. Web 2.0 systems have great potential to enhance health information and open new ways for patients and practitioners to communicate.

**Objective:**

To develop a Web portal designed to facilitate self-management, including diabetes-related information and social networking functions, and to study its use and effects in pediatric patients with diabetes.

**Methods:**

A Web 2.0 portal was developed in collaboration with patients, parents, and practitioners. It offered communication with local practitioners, interaction with peers, and access to relevant information and services. Children and adolescents with diabetes in a geographic population of two pediatric clinics in Sweden were randomized to a group receiving passwords for access to the portal or a control group with no access (n=230) for 1 year. All subjects had access during a second study year. Users’ activity was logged by site and page visits. Health-related quality of life (HRQOL), empowerment (DES), and quality of information (QPP) questionnaires were given at baseline and after 1 and 2 study years. Clinical data came from the Swedish pediatric diabetes quality registry SWEDIABKIDS.

**Results:**

There was a continuous flow of site visits, decreasing in summer and Christmas periods. In 119/233 families (51%), someone visited the portal the first study year and 169/484 (35%) the second study year. The outcome variables did not differ between intervention and control group. No adverse treatment or self-care effects were identified. A higher proportion of mothers compared to fathers visited once or more the first year (*P*<.001) and the second year (*P*<.001). The patients who had someone in the family visiting the portal 5 times or more, had shorter diabetes duration (*P*=.006), were younger (*P*=.008), had lower HbA1c after 1 year of access *(P=*.010), and were more often girls *(P<*.001). Peer interaction seems to be a valued aspect.

**Conclusions:**

The Web 2.0 portal may be useful as a complement to traditional care for this target group. Widespread use of a portal would need integration in routine care and promotion by diabetes team members.

**Trial Registration:**

International Standard Randomized Controlled Trial Number (ISRCTN):92107365; http://www.controlled-trials.com/ISRCTN92107365/ (Archived by WebCite at http://webcitation.org/6IkiIvtSb).

## Introduction

Diabetes requires extensive self-care and comprehensive knowledge. The management of the disease, including insulin injections and self-control of blood glucose, affects everyday life, thus coping skills are essential. Health-related quality of life (HRQOL) may be influenced, particularly diabetes-related influence on HRQOL [1-3]. The association between good metabolic control and risk reduction for late complications is known [4-6] but despite modern treatment, only one third of the patients reach treatment target [7,8]. Efforts to increase patients’ and parents’ knowledge are needed to empower them in their self-care [9].

Thus patient education is central to diabetes self-management [10]. Studies in adult type 1 diabetes populations have indicated that structured patient training and education as part of intensive treatment reduces HbA1c with no increase in severe hypoglycemia, or even with persistent reduction of severe hypoglycemia [11-14]. Although such findings are consistent with modern clinical practice and experience [15], evidence repeatedly has been found insufficient to recommend adaptation of any particular educational method or program for type 1 diabetes [16,17]. There are several approaches, but there is no single one that emerges as clearly dominant.

We previously found that in pediatric patients’ and parents’ perspective on quality of care, improvements are needed regarding information and access to services [18]. In a multinational study, receiving information at diagnosis and having access to multiple sources of information later on have been associated with better outcomes from patients’ and parents’ perspectives [19]. The most frequently used sources of information, both for young adults and parents with diabetes, were diabetes medical teams, websites, and diabetes associations, with the diabetes team being the main source.

Social support is important for psychosocial adaptation when living with a pediatric chronic disease [20]. Recent research demonstrates how online support groups may contribute to patient empowerment [21]. Empowering processes identified among adult users of online support groups include exchanging information, encountering emotional support, finding recognition, sharing experiences, helping others, and amusement [21,22].

The Internet is a rapidly emerging source of health services and information [23]. Most adolescents and young adults find it convenient to use the Internet to communicate and find information, including on health topics [24], but still students lack knowledge about searching and evaluating health information on the Internet [25]. The umbrella term “Web 2.0” describes a range of widely used Internet applications to enhance participation, collaboration, openness, social networking, and peers’ sharing information [26]. Web 2.0 systems have great potential to enhance health information delivery and exchange whenever and wherever it is needed, including use of new mobile devices.

Use of new information and communication technologies show promise regarding improved diabetes care in general [27-32]. At least those patients with poor metabolic control, greater use of health care services, higher motivation and/or less experience with diabetes treatment seem to benefit, although few significant long-term effects on main outcomes have been shown. Positive effects on knowledge and psychosocial well-being have been found as a result of Internet educational interventions in adolescents with diabetes [33].

Adolescents with a chronic health problem have been found to respond positively to sites that target them and their needs, including focused chat rooms and message boards [34]. In a pilot study, an Internet-based system aimed at assisting in diabetes management was found to be feasible and well accepted but did not influence HRQOL or metabolic control [35]. In a related study, communication with peers seemed to improve much more than their communication with practitioners [36]. Adolescents with diabetes visited various online forums for social support, information, advice, and shared experience [37]. Females used discussion forums more frequently and males requested more information.

In our study, we hypothesized that a Web 2.0 portal, with diabetes-related information and the possibility to communicate with diabetes peers as well as with health care professionals, would (1) be used, (2) be of complementary value in everyday life with diabetes, especially by newly diagnosed patients and patients in periods with instable metabolic control, (3) be perceived as helpful in self-treatment, and (4) contribute to improved metabolic control.

Thus we aimed to develop a Web portal designed to facilitate self-management, with diabetes-related information and the possibility to communicate with others with diabetes and health care professionals, and to study the use and its effects in pediatric patients with diabetes and their parents.

## Methods

### Ethics Statement

The study was approved by the Research Ethics Committee of the Faculty of Health Science at Linköping University, Sweden. Basic information about the study was given to adolescents and parents by posted letters. They were informed in the letter about confidentiality and the right to withdraw without explanation. All participants including next of kin were required to return a signed consent form. Informed consent was also given by each participant in electronic form prior to the first visit to the portal.

This study was a randomized controlled trial (ISRCTN92107365) and the CONSORT checklist is available as supporting information (see Multimedia Appendix 1).

### The Portal

The Diabit Web 2.0 portal, as described elsewhere [38], offered self-directed communication with health professionals, interaction with peers, and access to information. The portal had been developed through a user-centred design process that included iterative sessions with groups of patients and parents as well as with the involved diabetes teams [39,40]. A prototype was piloted in 2005, and the portal Diabit was launched in April 2006. The portal was designed for complementary use by pediatric patients, parents, and practitioners whenever needed and by users’ own initiative.

**Figure 1:**
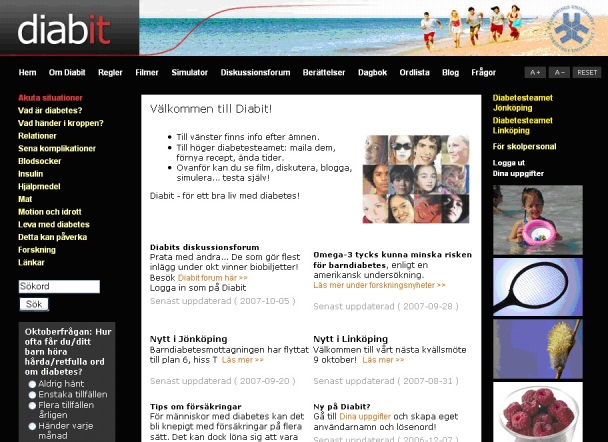
Screenshot from the Diabit portal. Home ”Welcome to Diabit”.

**Figure 2:**
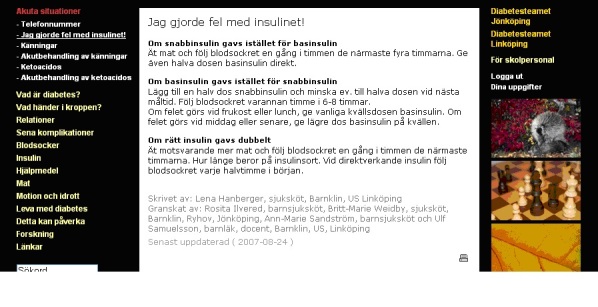
Screenshot from the Diabit portal. “I made a mistake with the insulin”, and how to manage.

**Figure 3:**
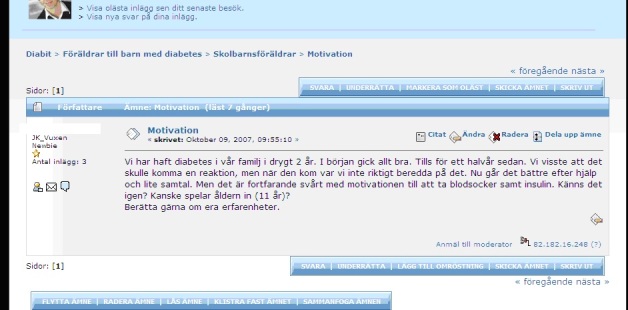
Screenshot from the Diabit portal. Discussion board.

It contained specific diabetes-related information and social networking functions such as a storyboard, a simple blog module, and discussion board modules (see examples of user interface in Figures 1-3). The discussion board in this version of the portal was designed for peer communication only (with safety issues monitored by a passive pediatrician). The practitioners involved did not have access to the patients’ discussion board, and parents had no access to adolescents’ board and vice versa.

Extensive information was given in text pages on essential areas of diabetes and in videos, and there was interactive simulator software as well [41]. Specific diabetes-related information on 13 main topics, developed by sessions with patients, and divided into 99 subtopics/Web pages, was written by team members. Links on diabetes-related information were: Acute situations, What is diabetes, Relations, Late complications, Insulin, Devices, Food, Blood glucose, Exercise and sports, Living with diabetes, This can affect, Research, and External links. Each section was revised by other team members from the two hospitals.

The portal also provided services for medical prescription renewal, appointments, and open questions and other general information about the local diabetes teams and their services. In addition, each respective group of professionals comprising the two local diabetes teams summarized important basic information using a personal tone when expressing, “What I may say to newly diagnosed children and their parents”.

To stimulate new visits, there was a function to highlight local practitioners’ news and information about local activities, and for new information in the areas of research, nutrition, devices, and others. Quarterly newsletters were sent linking to the news, and flyers were sent yearly with regular post to patients.

### Protocol and Assignment

**Figure 4:**
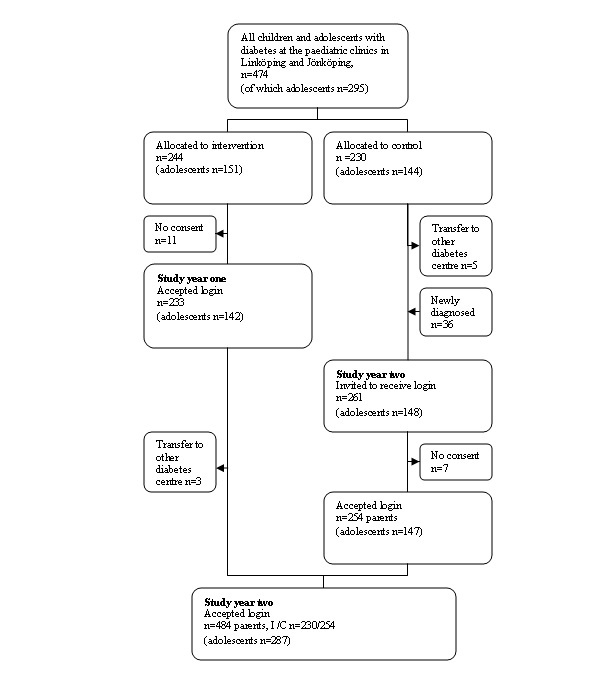
Flow chart of the intervention and the control group.

For inclusion, all the clinically diagnosed type 1 diabetes children, aged 0-18 years, registered in the Swedish pediatric diabetes quality registry, SWEDIABKIDS, belonging to the geographic population of the two pediatric clinics in Linköping and Jönköping, were eligible and invited to the study (Figure 4). The two clinics treated all young type 1 diabetes patients in their catchment areas. The patients and their families were randomized (stratified for clinic) by two of the authors (SN, LH), using a table of random numbers, to either the intervention group or the control group (Figure 4).

At baseline April 2006, all subjects in the intervention group were offered a personal password to the portal for the first year of the study. After study year 1, all subjects in the previous control group were also offered passwords to the portal (Figure 4). For children 13 years of age and older, both parents and adolescents received passwords while for younger children, only parents received passwords.

As shown in Figure 4, for the first study year, 233 patients and their parents (adolescents n=142) accepted, and in the second study year, an additional 254 patients and their parents (adolescents n=147) from the previous control group accepted as well. All diabetes team members of both hospitals (n=28) received a personal password as well at baseline. During the study, there were no directions of use given to patients and parents from any other part, and it was not related to any structured education activity.

### Masking

All personnel were blinded to group assignment for the first study year. Care providers took part in development of the portal, as described above, and were informed about the study but had no information on assignment. They were instructed to discuss any clinical issue raised by the patient as usual, without trying to identify the group to which the patient belonged.

### Clinical Characteristics and Diabetes Treatment

The groups were equal regarding baseline clinical characteristics (Table 1). In Sweden, diabetes teams at pediatric clinics, consisting of diabetes specialist nurses, registered nurses, diabetes specialist physicians, dieticians, social workers and/or clinical psychologists, treat all patients in the catchment area. The treatment policy was multiple insulin therapy. The member of the diabetes team encouraged active self-control and offered psychosocial support and problem-based education. The process of care and treatment policy has been described elsewhere [15,42].

**Table 1:** Characteristics of the population at baseline, intervention and control group, and most recent HbA1c at baseline (2006) and after of study year 1 (2007).

		Intervention group n=244	Control group n=230	*P* value
Sex (F/M), proportions, %		52/48	51/49	.82
Mean age, years (range, SD)		13.2 (2.8-18.5, 3.7)	13.3 (3.0-18.5, 3.7)	.66
Age at diagnosis, years, mean (range, SD)		8.1 (1-17, 3.7)	7.8 (1-16, 4.2)	.50
Duration, years, mean (range, SD)		4.9 (0.1-17.7, 3.7)	5.1 (0.1-14.6, 3.7)	.67
HbA1c baseline, %, mean (range, SD)		6.8 (4.2-11.4, 1.2)	6.8 (4.3-12.5, 1.2)	.91
HbA1c after study year 1, %, mean (range, SD)		6.7 (4.1-13.0, 1.2)	6.7 (3.7-10.8, 1.2)	.72
Insulin dose, U/kg/day, mean (range, SD)		1.0 (0.2-2.1, 0.3)	1.0 (0.3-2.0, 0.3)	.95
CSII^a^, proportion, %		16	16	1.00
# of injections/day, mean (range, SD)		5.3 (2-9, 1.0)	5.4 (2-10, 1.0)	.37
# of self controls/day, mean (range, SD)		4.4 (0.5-12.5, 2.3)	4.1 (0-10, 1.7)	.51
**Hypo last 12 months, proportion, %**				
	Needing help	16	25	.12
	Unconsciousness	4	4	.71
Comorbidity		18	20	.16

^a^CSII: continuous subcutaneous insulin infusion.

### Study Period

The first study period, year 1, was defined as April 11, 2006, to September 25, 2007. The planned 12-month study period was extended due to initially slow inclusion of active users. The second study period, year 2, was defined as September 26, 2007, to September 25, 2008. However, there is a lack of logged data from August 30, 2008, due to problems with the data server.

### Process Data

Logged data from the systems server were used to study frequencies and temporal patterns of patients and parents, as well as their practitioners, site visits, and page hits of the portal.

### Outcome Variables

Effects on HRQOL, empowerment, and perception of quality of care regarding information were measured and obtained from postal surveys. The clinical variables measured were HbA1c (data received from the Swedish pediatric diabetes quality registry, SWEDIABKIDS) [43], numbers of severe hypoglycemia (self-reported), and numbers of self-controls of blood glucose (self-reported).

### Questionnaires

For HRQOL, we used the DISABKIDS chronic-generic module, short form (12 items), adolescent, and parent (as proxy) version combined with the diabetes-specific module (10 items), adolescent and parent (as proxy) version [44,45]. The items in the chronic-generic module were assigned to six dimensions: independence, emotion, social inclusion, social exclusion, limitation, and medication. The items in the diabetes-specific module were assigned to treatment and impact on a 5-point Likert scale, where a low value corresponds to low quality of life.

Quality of care, regarding information, was measured by using selected questions from the Quality from the Patients’ Perspective (QPP) questionnaire [46,47]. The QPP instrument was developed using grounded theory. The items evaluate both perceived reality of the care received and for subjective importance of that particular item, for example, “I get sufficient information regarding insulin pen/pump. A. This is how it is for me. B. This is how important it is for me.” A 4-point scale ranging from “Fully agree” to “Do not agree at all” is used.

Empowerment was assessed by the Swedish Diabetes Empowerment Scale, short version (SWE-DES-SF-10) [48]. It includes four empowerment subscales: goal achievement, self-awareness, stress management, and readiness to change. A 5-point Likert scale is used.

Questions about access and using habits of the Internet, also used by Statistics Sweden [49] were included as well as a range of treatment-related questions and questions on socioeconomy, frequency of contact with peers, as well as online diabetes information search experiences.

Adolescents and parents completed questionnaires before baseline, posted late January 2006 (243 girls, 231 boys); after study year 1, posted late August 2007 (253 girls, 241 boys); and year 2, posted late August 2008 (250 girls, 234 boys) respectively. A mailed questionnaire and a stamped return envelope, with two subsequent reminders, were sent to all parents from an independent department at Linköping University. The response rates of the questionnaires were, in parents and adolescents respectively, at baseline 70% and 63%, after study year 1, 62% and 50%, and after study year 2, 59% and 65%.

### Clinical Variables

Data from the Swedish pediatric diabetes quality registry, SWEDIABKIDS [35] were used regarding diabetes duration, hemoglobin A1c (HbA1c), and insulin dose. Swedish HbA1c values were approximately 1% lower than DCCT/National Glycohemoglobin Standardization Program (NGSP) values [50]. Local routine methods for HbA1c determination, calibrated to the national standard method Mono-S, were utilized.

### Analysis

As a randomized control study, we compared the intervention and the control group at baseline and after study year 1. Additionally in both groups separately, baseline data were compared to data after study years 1 and 2. Most recent HbA1c values for each patient at baseline, at the end of study year 1 and at the end of study year 2 were used.

### Active User Analysis

In a separate analysis before and after the first year of access, active users were defined as those where someone in the family logged in five times or more during their first year with access to the portal. This cut-off level for active use was defined retrospectively taking into account the distribution of frequency of use.

The group of active users were compared to those with zero to four site visits during the same time period. Thus we merged data for the intervention group at baseline and after 1 year only (study year 1), and for the previous control group before and after 1 year of access respectively (study year 2).

### Statistical Methods

Summing the raw scores of the items in DISABKIDS representing each domain and dividing by the answers in a domain (at least 5/6 or 4/5 answers in each domain are required) resulted in mean domain scores. Grand mean of generic and diabetes-specific HRQOL was derived from summing the item mean score and dividing by the numbers of items. The scale for generic and diabetes-specific HRQOL was converted to a scale of 0-100, where 0 corresponds to 1 on the 5-point scale and 100 corresponds to 5. As primary endpoints for HRQOL, we used the mean of generic and diabetes-specific HRQOL and the mean of the dimensions within these. Total scale Swe-DES-SF-10 was calculated by summing the ten items and dividing by 10.

For comparisons, Mann-Whitney U test and Wilcoxon signed rank test were used and when data were normally distributed Student’s *t* test, paired and unpaired was used. On categorical variables, Chi-square test was used. *P* values <.05 were regarded as significant. Mean and SD are given. For statistical analysis, SPSS 17.0 software was used.

## Results

### Overview

No differences in the baseline characteristics of the population were found between the intervention and control group (Table 1). No differences was found regarding socioeconomics, access to the Internet, or information search and peer contacts.

### Use of the Portal

**Figure 5:**
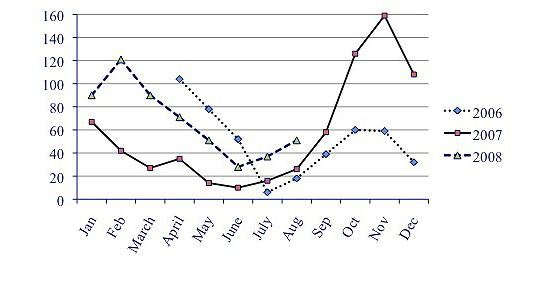
Site visits per month by patients and parents (study year 2 started 2007).

During the very first month after launch, 51 users (14 adolescents, 26 mothers, 11 fathers) from 39 families of the intervention group visited the portal once or more (1456 page visits). The long-term pattern indicated a continuous interest for site visits, decreasing during summer and Christmas periods, as shown in Figure 5 (similar pattern for numbers of page visits and visitors, data not shown).

During the first study year, 159 users made 695 visits to the portal (adolescents 163, mothers 363 and fathers 169), mean 4.4 visits, range 1-45, median 2, and 6421 page hits (adolescents 1611, mothers 3484 and fathers 1326), mean 39.2, range 1-330, median 28.

During the second study year, 207 users made 980 visits (adolescents 210, mothers 573, and fathers 197), mean 4.7 visits, range 1-132, median 2, and 5940 page hits (adolescents 1954, mothers 3364, and fathers 622), mean 28.7, range 1-381, median 20. Thus the mean numbers of page visits per site visit in study year 1 was 9.2 (by adolescents 9.9, mothers 9.6, and fathers 7.8) and in study year 2, 6.1 (by adolescents 9.3, mothers 5.9, and fathers 3.0) respectively.

**Figure 6:**
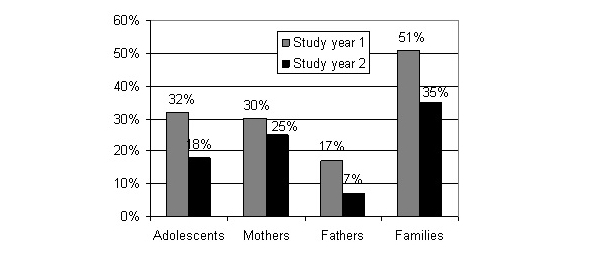
Proportions of adolescents, mothers, fathers, or at least one of these in the same family, logged in once or more.

The proportions of those visiting the portal at least once or more during study year 1 and 2 respectively are shown in Figure 6, with higher proportions of mothers as compared to fathers the first (*P*<.001) and the second study year (*P*<.001). Out of those patients where someone in the family visited at least once during study year 1 (n=119, 51%) and year 2 (n=169, 35%) respectively, the proportions of active users (five times or more) were 30% the first study year and 64% the second study year.

More frequent page hits were seen during the first study year on social networking with peers such as Blogs and Stories followed by Questions answered by the diabetes team as well as their News and updates (Table 2). This pattern was largely similar during study year 2 (data not shown).

**Table 2:** Page hits on frequently visited pages, intervention group study year 1.

Webpages	Mothers n=71		Fathers n=39		Adolescents n=46	
	Hits	%	Hits	%	Hits	%
Home	358	10.4	168	12.7	163	10.1
Stories^a^	227	6.6	85	6.4	101	5.3
Blogs^a^	178	5.2	93	7.1	120	7.5
Team Jönköping	114	3.3	53	4.0	25	1.6
Questions and answers	109	3.1	17	1.3	29	1.8
Team Linköping	76	2.2	35	2.6	34	2.1
Research	75	2.2	38	2.9	15	.9
Simulator	62	1.8	36	2.7	37	2.3
Food	58	1.7	18	1.4		
This can affect	51	1.5	17	1.3	24	1.5
Devices	49	1.4	31	2.3		
Living with diabetes	43	1.2			26	1.6
Late complications	36	1.0	15	1.1	19	1.2
Videos	34	1.0	27	2.0	44	2.7
Discussion board^a^	34	1.0	12	.9	20	1.2
External links	32	.9				
Relations	30	.9	12	.9	20	1.2

^a^Social networking.

### The Intervention Group Compared to the Control Group

No differences were found at baseline and after study year 1 between the intervention and control group, adolescents and parents respectively, regarding the outcome variables (HRQOL, empowerment, perception of quality of care regarding information measured by DISABKIDS, SWE-DES-SF-10, and QPP respectively, HbA1c, severe hypoglycemia, frequency of blood glucose self-control). No differences were found at baseline and after study year 1 and 2 respectively, neither in the intervention group nor in the control group, for adolescents and parents respectively, regarding the same outcome variables.

### Active Users

During the first year of access to the portal, active users (families where someone made five visits or more, n=68), compared to the less active (0-4 visits respectively, n=419) were younger, had shorter durations, and had lower HbA1c after 1 year, but there was only a moderate—not significant—change in the HbA1c difference over time (Table 3). No differences were found among active users at 1 year compared to baseline regarding the questionnaires and clinical outcome variables. A higher proportion of girls’ families were found among the active users (*P*<.001). A similar proportion of active users 7/68 (10%) had HbA1c >8 as in the comparison group, 56/419 (13%) (pediatric .535).

### Practitioners’ Use

A total of 20 users out of the 28 diabetes team members who received a password made 459 site visits during study year 1 and 2552 page visits, mean 5.6 page visits per site visit*,* range 3-370. In study year 2, they made 426 site visits and 1712 page visits, mean 4.0 page visits per site visit, range 6-826. Out of the 28 receiving passwords, 16 made ≥5 visits (15 users study year 1, 9 users study year 2).

**Table 3:** Active users (5 visits or more) as compared to less active (0-4 visits), including most recent HbA1c before access and at the end of 1 year of access.

	Active users	Comparison group	*P* value
Sex, % girls	65	49	<.001
Age, years, mean (range, SD)	12.1 (3.2-18.3, 3.8)	14.0 (1.9-18.5, 3.8)	.008
Duration, years, mean (range, SD)	3.9 (0.1-14.6, 3.3)	5.5 (0.1-17.7, 3.8)	.006
HbA1c before, %, mean (range, SD)	6.5 (3.9-9.0, 1.1)	6.8 (3.7 -11.4, 1.3)	.056
HbA1c at 1 year, %, mean (range, SD)	6.4 (4.2-10.10, 1.1)	6.8 (4.1-14.0, 1.2)	.010

## Discussion

### Principal Findings

The portal attracted all groups of users with great individual variation in frequency of use, and as expected, not all were users. In contrast to research on structured patient education including Web 2.0, this study did not evaluate a directed intervention program. The portal was a complementary information and communication resource for self-directed use whenever needed. Based on known information needs, developed through patient, parent, and health practitioners’ interaction, the portal merely offered a practitioner-driven high-quality alternative for online information and communication. Thus we performed this experiment as close to a future real-world resource as possible, leaving it to patients and parents to make their own decisions on use of the portal. In contrast to numerous other online resources though, the patients’ local multiprofessional pediatric diabetes teams created and/or verified the information contents.

The active users’ data seem somewhat promising, although the overall usage rate was low. As expected, a higher frequency of use was related to shorter diabetes experience. The frequency of use might also relate to factors such as personal interest and motivation, and/or perceived health status and satisfaction with the traditional care and education. For clinicians, it is a challenge to motivate and support young patients struggling with impaired metabolic control and this group must be given high priority for efforts. For patients reaching the HbA1c 8% level, interventions in our clinic are monthly visits, intensified adjustment of treatment, use of glucose sensors, and/or referral to the ward. As such patients were equally found among the active users, the portal appears useful also for this group of patients and their parents. This is in line with earlier findings that patients with poor metabolic control seem to benefit, sometimes even more than others, from the use of electronic communication [30].

In our qualitative evaluation presented elsewhere, users found the portal widely useful with the combination of peer support, reliable facts, and updates [38]. Being enabled to search when necessary and find reliable information provided by local clinicians was regarded as a great advantage, facilitating a feeling of security and being in control. Finding answers to difficult-to-ask questions, questions users did not know they had before, and questions focusing on sensitive areas were important. Visiting the portal could generate more information than expected, which could inspire increased use, and many had high positive expectations of a larger patient community. However, the password requirement appeared to be a real obstacle for some, probably limiting the number of users as well as frequency of use.

Already in the first study year, a high proportion of page hits on interactive functions indicated great interest in communication with peers and also for users’ questions answered by practitioners (Table 2) consistent with other findings [51]. Adolescent users and parents as well submitted stories, blogs, and comments increasingly during both years. From the second study year, a lively discussion board was started by parents, whereas the adolescent discussion board remained largely silent both study years. The discussion board privacy concept with access only for the peer group involved did not prove advantageous, but many parents appreciated their board as such.

As reported elsewhere, although many participants submit few or no posts, the community attracts a high proportion of site visits and many derive passive support from reading about the experiences of others, both positive and negative [36,38]. Thus we believe an open access discussion board is more likely to create benefit for many, along with the development and impact of Web 2.0 towards openness [26]. Further, our data confirm previous findings that girls and mothers seem more active in searching for information and more eager to communicate electronically [32,37,52], and we also found a notable lower rate of boys’ families among the active users. Further research in this area will need to be sensitive to gender differences.

### Strengths and Limitations

Some strengths of this study are a large study base and the controlled design including randomization of patients to either the intervention or the control group. The development of the portal with parents, adolescents, and diabetes team members participating laid the foundation for an appealing portal and a user-friendly design. No undesired effects were found on self-perceived HRQOL, empowerment, and metabolic control, which happened in some intervention programs [53]. No incidents of any undesired treatment effects related to the portal use were reported from practitioners or patients.

The overall absence of statistically significant effects should be interpreted with caution because (1) the number of active users in the intervention group was low, (2) there was no promotion from the blinded practitioners in the randomized controlled phase, and (3) frequencies of use were limited by a password requirement having the function of a gatekeeper [38]. A limitation of the study is that the effect on patient and parent knowledge of diabetes was not evaluated.

Also during the second study year, in spite of positive attitudes over a long-term involvement in the development and contents [38,40], many practitioners had a hard time starting to make use of the portal in their practice (unpublished data). Various obstacles were reported, such as deep-rooted habits, having no computer in the room, or having too many working tasks. An important issue is how to increase engagement in patients and their next of kin. Whereas this study cannot answer this, practitioners’ views recently have been further explored [54].

The Internet remains a rather new tool in patient education, and the implementation of using it is not a rapid process in routine care [38,40]. For practitioners trained in a culture of care with secrecy and strong restrictions regarding dissemination of patients’ data, the global process towards openly sharing personal health information on the Internet [55] initially might seem somewhat uncomfortable and confusing. However, provided that practitioners can control and/or monitor the information content in the portal, over time their motivation for using it in their daily practice will probably increase.

During the second study year (second year of access for the intervention group), the overall proportion of the study participants who used the portal decreased, as some previous intervention group users presumably decreased their use after a longer time (Figure 6). Also in other interventions with Internet-based systems, a decline in use over time has been found [35,36]. In implementation of Web 2.0 systems for patients, attention should be paid to highlighting the feed of new information from their practitioners, as well as new messages and blogs posted from peer users, and strategies for external advertisements and reminders are needed as well [38].

To sum up, the logged user behaviors and our qualitative evaluations indicate that a fully implemented Web 2.0 system including a larger population for a community and without passwords might be of great complementary value for both patients and professionals [38]. Future research also involving larger sample sizes and with multicentre collaboration might add knowledge on development of various effective educational interventions [17]. Patient engagement and social marketing of new tools warrant more attention.

Following this study in subsequent scientific experiments, the Diabit Web 2.0 portal was rebuilt and opened for free use on the open Internet, including an open discussion board, on World Diabetes Day November 14, 2008. The total user rate increased in 2009 to 29,015 yearly visits (144,336 page visits) from all over the nation, showing a continuous interest for both the growing community as well as factual information.

### Conclusions

This study supports the fact that a Web 2.0 portal may be successfully used as a complement to traditional patient education and support. The implementation might be further enhanced by easy access without passwords, by highlighting new information, by active promotion from active diabetes team members and through other reminders in the structure of care. Future research on electronic communication targeting young people with long-term health problems will need to focus more on use of Web 2.0, including gender aspects.

## Multimedia Appendix 1

CONSORT-EHEALTH checklist V1.6.2 [56].

## Abbreviations

CSII: subcutaneous continuous insulin infusion
DCCT: Diabetes Control and Complications Trial Research Group
DES: diabetes empowerment scale
HbA1c: hemoglobin A1c
HRQOL: health-related quality of life
NGSP: National Glycohemoglobin Standardization Program
QPP: quality from the patients’ perspective
SWEDIABKIDS: Swedish pediatric diabetes quality registry
SWE-DES-SF-10: Swedish diabetes empowerment scale, short version
