# Supplementary material for: Use of a Web 2.0 Portal to Improve Education and Communication in Young Patients With Families: Randomized Controlled Trial
Source: J Med Internet Res. 2013 Aug 23;15(8):e175. doi: 10.2196/jmir.2425 (PMC3758041; doi:10.2196/jmir.2425)
Supplement: Supplementary file 1 [file jmir_v15i8e175_app1.pdf]

CONSORT-EHEALTH Checklist V1.6.2 Report

(based on CONSORT-EHEALTH V1.6), available at [http://tinyurl.com/consort-ehealth-v1-6].

2425

Date completed

6/12/2013 11:38:43

by

Lena Hanberger

USE OF A WEB 2.0 PORTAL TO IMPROVE EDUCATION AND COMMUNICATION IN PAEDIATRIC DIABETES PATIENTS WITH FAMILIES. A RANDOMISED CONTROLLED TRIAL

TITLE

1a-i) Identify the mode of delivery in the title

The title is:
"Use of a web 2.0 portal to improve education and communication in young patients with families. A randomised controlled trial."
(Mode of delivery: web 2.0 portal)

1a-ii) Non-web-based components or important co-interventions in title

No other interventions were performed.

1a-iii) Primary condition or target group in the title

The title is:
"Use of a web 2.0 portal to improve education and communication in young patients with families. A randomised controlled trial."
(Target group: Young patients with families)

ABSTRACT

1b-i) Key features/functionalities/components of the intervention and comparator in the METHODS section of the ABSTRACT

" It offered communication with local practitioners, interaction with peers and access to relevant information and services."

1b-ii) Level of human involvement in the METHODS section of the ABSTRACT

"A Web 2.0 portal was developed in collaboration with patients, parents and practitioners."

"Children and adolescents with diabetes in a geographic population of two paediatric clinics in Sweden were randomized to a group (n=233) receiving passwords for access to the portal, or a control group with no access (n=230) for one year."

1b-iii) Open vs. closed, web-based (self-assessment) vs. face-to-face assessments in the METHODS section of the ABSTRACT

"Children and adolescents with diabetes in a geographic population of two paediatric clinics in Sweden were randomized to a group (n=233) receiving passwords for access to the portal, or a control group with no access (n=230) for one year. "
"Measures: Users activity logged by site and page visits. Health-related quality of life (HRQOL), empowerment (DES), and quality of information (QPP) questionnaires at baseline, after one and two study years. Clinical data from the Swedish paediatric diabetes quality registry SWEDIABKIDS."

1b-iv) RESULTS section in abstract must contain use data

"Children and adolescents with diabetes in a geographic population of two paediatric clinics in Sweden were randomized to a group (n=233) receiving passwords for access to the portal, or a control group with no access (n=230) for one year. "
"There was a continuous flow of site visits, decreasing in summer and Christmas periods. In 119/233 families (51%) someone visited the portal the first study year and in 169/484 (35%) the second study year. The outcome variables did not differ between intervention and control group. No adverse treatment or self-care effects were identified. A higher proportion of mothers compared to fathers visited once or more the first (p< .001) and the second year (P<0.001). The patients where someone in the family visited the portal five times or more, had shorter diabetes duration (P= .006), were younger (P= .008), had lower HbA1c after one year of access (P= .010), and were more often girls (P< .001). Peer interaction seems to be a valued aspect"

1b-v) CONCLUSIONS/DISCUSSION in abstract for negative trials

"The Web 2.0 portal may be useful as a complement to traditional care for this target group. Widespread use of a portal probably needs integration in routine care and promotion by diabetes team members. "

INTRODUCTION

2a-i) Problem and the type of system/solution

Incorporated in a broader health program: "be of complementary value in everyday life with diabetes,"
Intented for a particualr population: "...and study the use and its effects in paediatric patients with diabetes and their parents. "
Goals of intervention: "...we aimed to develop a Web portal designed to facilitate self-management, with diabetes-related information and the possibility to communicate with others with diabetes and health care professionals,"

2a-ii) Scientific background, rationale: What is known about the (type of) system

"Use of new information and communication technologies show promise regarding improved diabetes care in general "
"Adolescents with a chronic health problem have been found positive to sites that target them and their needs, including focused chat rooms and message boards "
"Adolescents with diabetes visit various online forums for social support, information, advice, and shared experience [37]. Females use discussion forums more frequently and males request more information. "

METHODS

3a) CONSORT: Description of trial design (such as parallel, factorial) including allocation ratio

"In the present case study we hypothesised that a Web 2.0 portal, with diabetes-related information and the possibility to communicate with diabetes peers as well as with health care professionals, would (a) be used; b) be of complementary value in everyday life with diabetes, especially by newly diagnosed patients and patients in periods with instable metabolic control; (c) be perceived as helpful in self treatment; and (d) contribute to improved metabolic control.

Thus we aimed to develop a Web portal designed to facilitate self-management, with diabetes-related information and the possibility to communicate with others with diabetes and health care professionals, and study the use and its effects in paediatric patients with diabetes and their parents. "

3b) CONSORT: Important changes to methods after trial commencement (such as eligibility criteria), with reasons

Not Applicable.
No important changes were performed after the trial commencement.

3b-i) Bug fixes, Downtimes, Content Changes

Not Applicable.
No important changes were performed after the trial commencement.

4a) CONSORT: Eligibility criteria for participants

"For inclusion, all the clinically diagnosed type 1 diabetes children, aged 0-18 years, registered in the Swedish paediatric diabetes quality registry, SWEDIABKIDS, belonging to the geographic population of the two paediatric clinics in Linköping and Jönköping, were eligible and invited to the study (Figure 4). The two clinics treated all young type 1 diabetes patients in their catchment areas."

4a-i) Computer / Internet literacy

From the introduction section:

" Informed consent was also given by each participant in electronic form prior to the first visit to the portal."

"The most frequently used sources of information, both for young adults and parents with diabetes, were diabetes medical teams, websites and diabetes associations,"

Computer /Internet literacy was not expressed as a eligibility criterion by the researchers but access to Internet was a prerequisite for participating.

4a-ii) Open vs. closed, web-based vs. face-to-face assessments:

"Basic information to adolescents and parents about the study was given by posted letters. They were informed in the letter about confidentiality and the right to withdraw without explanation. All participants including next of kin were required to return a signed consent form. Informed consent was also given by each participant in electronic form prior to the first visit to the portal."

"All personnel were blinded to group assignment for the first study year. Care providers took part in development of the portal, as described above, and were informed about the study but had no information on assignment. They were instructed to discuss any clinical issue raised by the patient as usual, without trying to identify the group to which the patient belonged."

4a-iii) Information giving during recruitment

"Basic information to adolescents and parents about the study was given by posted letters. They were informed in the letter about confidentiality and the right to withdraw without explanation. All participants including next of kin were required to return a signed consent form. Informed consent was also given by each participant in electronic form prior to the first visit to the portal."

"All personnel were blinded to group assignment for the first study year. Care providers took part in development of the portal, as described above, and were informed about the study but had no information on assignment. They were instructed to discuss any clinical issue raised by the patient as usual, without trying to identify the group to which the patient belonged."

4b) CONSORT: Settings and locations where the data were collected

"For inclusion, all the clinically diagnosed type 1 diabetes children, aged 0-18 years, registered in the Swedish paediatric diabetes quality registry, SWEDIABKIDS, belonging to the geographic population of the two paediatric clinics in Linköping and Jönköping, were eligible and invited to the study (Figure 4). "

4b-i) Report if outcomes were (self-)assessed through online questionnaires

No online questionnnaires were used:

"A mailed questionnaire and a stamped return envelope, with two subsequent reminders, were sent to all parents from an independent department at Linköping University."

4b-ii) Report how institutional affiliations are displayed

The reearchers affiliation were presented when information about and invitaion to the study as and furthermore along with the mailed questionnaires.

5) CONSORT: Describe the interventions for each group with sufficient details to allow replication, including how and when they were actually administered

5-i) Mention names, credential, affiliations of the developers, sponsors, and owners

#The portal had been developed through a user-centred design process which included iterative sessions with groups of patients and parents as well as with the involved diabetes teams"

Thor Balkhed, Bildamedia, Jörgen Isberg, Keylab and Anders Larsson, IDA, Linköping University contributed to elements of the portal design, setup and/or maintenance for the study.

5-ii) Describe the history/development process

"The portal had been developed through a user-centred design process which included iterative sessions with groups of patients and parents as well as with the involved diabetes teams [39, 40]. "

5-iii) Revisions and updating

Not Applicable

5-iv) Quality assurance methods

"Extensive information was given in text pages on essential areas of diabetes and in videos, and there was interactive simulator software as well [41]. Specific diabetes-related information on 13 main topics, developed by sessions with patients, and divided into 99 subtopics/web pages, was written by team members."

"Each section was revised by other team members from the two hospitals. "

5-v) Ensure replicability by publishing the source code, and/or providing screenshots/screen-capture video, and/or providing flowcharts of the algorithms used

Screen shots: Fig 1 - 3

References are given with information about the developement of the portal:

"The Diabit Web 2.0 portal, as described elsewhere [38], offered self-directed communication with health professionals, interaction with peers and access to information. The portal had been developed through a user-centred design process which included iterative sessions with groups of patients and parents as well as with the involved diabetes teams [39, 40]."

5-vi) Digital preservation

http://www.diabit.se/

5-vii) Access

"At baseline April 2006, all subjects in the intervention group were offered a personal password to the portal for the first year of the study. After study year one, all subjects in the previous control group were also offered passwords to the portal (Figure 4). For children 13 years of age and older, both parents and adolescents received passwords while for younger children only parents received passwords. "

"Following this study in subsequent scientific experiment, the Diabit Web 2.0 portal was rebuilt and opened for free use on the open Internet, including an open discussion board, on World Diabetes Day November 14, 2008."

5-viii) Mode of delivery, features/functionalities/components of the intervention and comparator, and the theoretical framework

"The portal
The Diabit Web 2.0 portal, as described elsewhere [38], offered self-directed communication with health professionals, interaction with peers and access to information. The portal had been developed through a user-centred design process which included iterative sessions with groups of patients and parents as well as with the involved diabetes teams [39, 40]. A prototype was piloted in 2005 and the portal Diabit was launched in April 2006. The portal was designed for complementary use by paediatric patients, parents and practitioners whenever needed and by users' own initiative.

It contained specific diabetes-related information and social networking functions such as a storyboard, a simple blog module and discussion board modules (examples of user interface in figures 1-3). The discussion board in this version of the portal was designed for peer communication only (with safety issues monitored by a passive paediatrician). The practitioners involved did not have access to the patients' discussion board, and parents had no access to adolescents' board and vice versa.

Fig 1. Screenshot from the Diabit portal. Home "Welcome to Diabit"

Figure 2. Screenshot from the Diabit portal. "I made a mistake with the insulin", and how to manage.

Figure 3. Screenshot from the Diabit portal. Discussion board.

Extensive information was given in text pages on essential areas of diabetes and in videos, and there was interactive simulator software as well [41]. Specific diabetes-related information on 13 main topics, developed by sessions with patients, and divided into 99 subtopics/web pages, was written by team members. Links on diabetes-related information were: Acute situations, What is diabetes, Relations, Late complications, Insulin, Devices, Food, Blood glucose, Exercise and sports, Living with diabetes, This can affect, Research and External links. Each section was revised by other team members from the two hospitals.

The portal also provided services for medical prescription renewal, appointments and open questions and other general information about the local diabetes teams and their services. In addition, each respective group of professionals comprising the two local diabetes teams summarized important basic information using a personal tone when expressing, "What I may say to newly diagnosed children and their parents."

5-ix) Describe use parameters

No instruction about the timing for use were given to the participants.
"During the study, there were no directions of use given to patients and parents from any other part and it was not related to any structured education activity."
"Quarterly newsletters were sent linking to the news and flyers were sent yearly with regular post to patients"

5-x) Clarify the level of human involvement

"As shown in figure 4, for the first study year 233 patients and their parents (adolescents n=142) accepted, and the second study year an additional 254 patients and their parents (adolescents n=147) from the previous control group accepted as well. All diabetes team members of both hospitals (n=28) received a personal password as well at baseline. "

5-xi) Report any prompts/reminders used

"Quarterly newsletters were sent linking to the news and flyers were sent yearly with regular post to patients"
The personnel "were instructed to discuss any clinical issue raised by the patient as usual, without trying to identify the group to which the patient belonged"

5-xii) Describe any co-interventions (incl. training/support)

No co- interventions were performed.

6a) CONSORT: Completely defined pre-specified primary and secondary outcome measures, including how and when they were assessed

Process data
Logged data from the systems server were used to study frequencies and temporal patterns of patients' and parents', as well as their practitioners', site visits and page hits of the portal.

"Outcome variables
Effects on HRQOL, empowerment and perception of quality of care regarding information were measured and obtained from postal surveys. The clinical variables measured were HbA1c (data received from the Swedish paediatric diabetes quality registry, SWEDIABKIDS) [43], numbers of severe hypoglycaemia (self-reported), and numbers of self-controls of blood glucose (self-reported). "
"Adolescents and parents completed questionnaires before baseline, posted late January 2006 (243 girls, 231 boys), after study year one, posted late August 2007 (253 girls, 241 boys) and year two, posted late August 2008 (250 girls, 234 boys) respectively."
"As a randomised control study we compared the intervention and the control group at baseline and after study year one. Additionally in both groups separately, baseline data were compared to data after study year one and two. Most recent HbA1c values for each patient at baseline, at the end of study year one and at the end of study year 2 were used. "

6a-i) Online questionnaires: describe if they were validated for online use and apply CHERRIES items to describe how the questionnaires were designed/deployed

No online questionnaires were used.

6a-ii) Describe whether and how "use" (including intensity of use/dosage) was defined/measured/monitored

All visits were logged. Furhtermore:
"In a separate analysis before and after the first year of access, active users were defined as those where someone in the family logged in five times or more during their first year with access to the portal. This cut-off level for active use was defined retrospectively taking into account the distribution of frequency of use.
The group of active users were compared to those with zero to four site visits during the same time period. Thus we merged data for the intervention group at baseline and after one year only (study year one), and for the previous control group before and after one year of access respectively (study year two)."

6a-iii) Describe whether, how, and when qualitative feedback from participants was obtained

"The portal had been developed through a user-centred design process which included iterative sessions with groups of patients and parents as well as with the involved diabetes teams [39, 40]. A prototype was piloted in 2005 and the portal Diabit was launched in April 2006. "

6b) CONSORT: Any changes to trial outcomes after the trial commenced, with reasons

Not Applicable

7a) CONSORT: How sample size was determined
7a-i) Describe whether and how expected attrition was taken into account when calculating the sample size
"For inclusion, all the clinically diagnosed type 1 diabetes children, aged 0-18 years, registered in the Swedish paediatric diabetes quality registry, SWEDIABKIDS, belonging to the geographic population of the two paediatric clinics in Linköping and Jönköping, were eligible and invited to the study (Figure 4). "
7b) CONSORT: When applicable, explanation of any interim analyses and stopping guidelines
Not Applicable
8a) CONSORT: Method used to generate the random allocation sequence
"All diabetes team members of both hospitals (n=28) received a personal password as well at baseline. "
8b) CONSORT: Type of randomisation; details of any restriction (such as blocking and block size)
"The patients and their families were randomised (stratified for clinic) by two of the authors (SN, LH), using a table of random numbers, to either the intervention group or the control group (Figure 4). "
9) CONSORT: Mechanism used to implement the random allocation sequence (such as sequentially numbered containers), describing any steps taken to conceal the sequence until interventions were assigned
Not Applicable
10) CONSORT: Who generated the random allocation sequence, who enrolled participants, and who assigned participants to interventions
"The patients and their families were randomised (stratified for clinic) by two of the authors (SN, LH), using a table of random numbers, to either the intervention group or the control group (Figure 4). "
11a) CONSORT: Blinding - If done, who was blinded after assignment to interventions (for example, participants, care providers, those assessing outcomes) and how
11a-i) Specify who was blinded, and who wasn't
"Masking
All personnel were blinded to group assignment for the first study year. Care providers took part in development of the portal, as described above, and were informed about the study but had no information on assignment. They were instructed to discuss any clinical issue raised by the patient as usual, without trying to identify the group to which the patient belonged."
11a-ii) Discuss e.g., whether participants knew which intervention was the "intervention of interest" and which one was the "comparator"
"Masking
All personnel were blinded to group assignment for the first study year. Care providers took part in development of the portal, as described above, and were informed about the study but had no information on assignment. They were instructed to discuss any clinical issue raised by the patient as usual, without trying to identify the group to which the patient belonged."
11b) CONSORT: If relevant, description of the similarity of interventions
Not Applicable
12a) CONSORT: Statistical methods used to compare groups for primary and secondary outcomes
"Statistical methods
Summing the raw scores of the items in DISABKIDS representing each domain and dividing by the answers in a domain (at least 5/6 or 4/5 answers in each domain are required) resulted in mean domain scores. Grand mean of generic and diabetes-specific HRQOL was derived from summing the item mean score and dividing by the numbers of items. The scale for generic and diabetes specific HRQOL was converted to a scale of 0-100, where 0 corresponds to 1 on the five-point scale and 100 corresponds to 5. As primary endpoints for HRQOL we used the mean of generic and diabetes-specific HRQOL and the mean of the dimensions within these. Total scale Swe-DES-SF-10 was calculated by summing the ten items and dividing by 10.
For comparisons Mann-Whitney U test and Wilcoxon signed rank test were used and when data were normally distributed Student's t-test, paired and unpaired was used. On categorical variables Chi-square test was used. P-values < .05 were regarded as significant. Mean and ±SD are given. "
12a-i) Imputation techniques to deal with attrition / missing values
Drop-outs were not included in the analysis.
Non-users were included in the non-user group.
12b) CONSORT: Methods for additional analyses, such as subgroup analyses and adjusted analyses
"In a separate analysis before and after the first year of access, active users were defined as those where someone in the family logged in five times or more during their first year with access to the portal."

RESULTS

13a) CONSORT: For each group, the numbers of participants who were randomly assigned, received intended treatment, and were analysed for the primary outcome
"As shown in figure 4, for the first study year 233 patients and their parents (adolescents n=142) accepted, and the second study year an additional 254 patients and their parents (adolescents n=147) from the previous control group accepted as well. All diabetes team members of both hospitals (n=28) received a personal password as well at baseline. "
13b) CONSORT: For each group, losses and exclusions after randomisation, together with reasons
Refers to CONSORT Flow diagram and fig 4 in the manuscript.
13b-i) Attrition diagram
Refers to CONSORT Flow diagram and fig 4 in the manuscript.
14a) CONSORT: Dates defining the periods of recruitment and follow-up
"For inclusion, all the clinically diagnosed type 1 diabetes children, aged 0-18 years, registered in the Swedish paediatric diabetes quality registry, SWEDIABKIDS, belonging to the geographic population of the two paediatric clinics in Linköping and Jönköping, were eligible and invited to the study (Figure 4). The two clinics treated all young type 1 diabetes patients in their catchment areas. The patients and their families were randomised (stratified for clinic) by two of the authors (SN, LH), using a table of random numbers, to either the intervention group or the control group (Figure 4).

At baseline April 2006, all subjects in the intervention group were offered a personal password to the portal for the first year of the study. After study year one, all subjects in the previous control group were also offered passwords to the portal (Figure 4). "
"Study period
The first study period, year one, was defined as April 11, 2006 to September 25, 2007. The planned 12-month study period was extended due to initially slow inclusion of active users. The second study period, year two, was defined as September 26, 2007 to September 25, 2008."
14a-i) Indicate if critical "secular events" fell into the study period
Not Applicable
14b) CONSORT: Why the trial ended or was stopped (early)
The study was carried out but:
"The planned 12-month study period was extended due to initially slow inclusion of active user"
15) CONSORT: A table showing baseline demographic and clinical characteristics for each group

Refers to:

Table 1. Characteristics of the population at baseline, intervention and control group, and most recent HbA1c at baseline (2006) and after of study year one (2007).

15-i) Report demographics associated with digital divide issues

Refers to:

Table 1. Characteristics of the population at baseline, intervention and control group, and most recent HbA1c at baseline (2006) and after of study year one (2007).

Social-economic status, computer/Internet/ehealth literacy of the participants was not known.

16a) CONSORT: For each group, number of participants (denominator) included in each analysis and whether the analysis was by original assigned groups

16-i) Report multiple “denominators” and provide definitions

Table 2. Page hits on frequently visited pages, intervention group study year one, social networking bolded.
Table 3. Active users (5 visits or more) as compared to less active (0-4 visits), including most recent HbA1c before access and at the end of one year of access.
"Use of the portal
During the very first month after launch, 51 users (14 adolescents, 26 mothers, 11 fathers) from 39 families of the intervention group visited the portal once or more (1,456 page visits). The long-term pattern indicated a continuous interest for site visits, decreasing during summer and Christmas periods, as shown in Figure 6 (similar pattern for numbers of page visits and visitors, data not shown).
During the first study year, 159 users made 695 visits to the portal (adolescents 163, mothers 363 and fathers 169), mean 4.4 visits, range 1-45, median 2, and 6,421 page hits (adolescents 1,611, mothers 3,484 and fathers 1,326), mean 39.2, range 1-330, median 28.
During the second study year, 207 users made 980 visits (adolescents 210, mothers 573 and fathers 197), mean 4.7 visits, range 1-132, median 2, and 5,940 page hits (adolescents 1954, mothers 3364 and fathers 622), mean 28.7, range 1-381, median 20.
Thus the mean numbers of page visits per site visit in study year one was 9.2 (by adolescents 9.9, mothers 9.6 and fathers 7.8) and in study year two 6.1 (by adolescents 9.3, mothers 5.9 and fathers 3.0) respectively.
The proportions of those visiting the portal at least once or more during study year one and two respectively are shown in Figure 5, with higher proportions of mothers as compared to fathers the first (P< .001) and the second study year (P< .001). Out of those patients where someone in the family visited at least once during study year one (n=119, 51%) and year two (n=169, 35%) respectively, the proportions of active users (five times or more) were 30% the first study year and 64% the second study year. "

16-ii) Primary analysis should be intent-to-treat

Primary analysis was intent-to-treat
Secondary analyses: "In a separate analysis before and after the first year of access, active users were defined as those where someone in the family logged in five times or more during their first year with access to the portal. This cut-off level for active use was defined retrospectively taking into account the distribution of frequency of use."

17a) CONSORT: For each primary and secondary outcome, results for each group, and the estimated effect size and its precision (such as 95% confidence interval)

"The proportions of those visiting the portal at least once or more during study year one and two respectively are shown in Figure 5, with higher proportions of mothers as compared to fathers the first (P< .001) and the second study year (P< .001). Out of those patients where someone in the family visited at least once during study year one (n=119, 51%) and year two (n=169, 35%) respectively, the proportions of active users (five times or more) were 30% the first study year and 64% the second study year. "
"The intervention group compared to the control group
No differences were found at baseline and after study year one between the intervention and control group, adolescents and parents respectively, regarding the outcome variables (HRQOL, empowerment, perception of quality of care regarding information measured by DISABKIDS, SWE-DES-SF-10 and QPP respectively, HbA1c, severe hypoglycaemia, frequency of blood glucose self-control). No differences were found at baseline and after study year one and two respectively, neither in the intervention group nor in the control group, for adolescents and parents respectively, regarding the same outcome variables."

Table 3. Active users (5 visits or more) as compared to less active (0-4 visits), including most recent HbA1c before access and at the end of one year of access.

17a-i) Presentation of process outcomes such as metrics of use and intensity of use

"During the first study year, 159 users made 695 visits to the portal (adolescents 163, mothers 363 and fathers 169), mean 4.4 visits, range 1-45, median 2, and 6,421 page hits (adolescents 1,611, mothers 3,484 and fathers 1,326), mean 39.2, range 1-330, median 28.
During the second study year, 207 users made 980 visits (adolescents 210, mothers 573 and fathers 197), mean 4.7 visits, range 1-132, median 2, and 5,940 page hits (adolescents 1954, mothers 3364 and fathers 622), mean 28.7, range 1-381, median 20.
Thus the mean numbers of page visits per site visit in study year one was 9.2 (by adolescents 9.9, mothers 9.6 and fathers 7.8) and in study year two 6.1 (by adolescents 9.3, mothers 5.9 and fathers 3.0) respectively.
The proportions of those visiting the portal at least once or more during study year one and two respectively are shown in Figure 5, with higher proportions of mothers as compared to fathers the first (P< .001) and the second study year (P< .001). Out of those patients where someone in the family visited at least once during study year one (n=119, 51%) and year two (n=169, 35%) respectively, the proportions of active users (five times or more) were 30% the first study year and 64% the second study year. "

17b) CONSORT: For binary outcomes, presentation of both absolute and relative effect sizes is recommended

Not Applicable

18) CONSORT: Results of any other analyses performed, including subgroup analyses and adjusted analyses, distinguishing pre-specified from exploratory

"Active users
During the first year of access to the portal, active users (families where someone made five visits or more, n=68), compared to the less active (0-4 visits respectively, n=419) were younger, had shorter duration, and had lower HbA1c after one year, but there was only a moderate - not significant - change in the HbA1c difference over time (Table 3). No differences were found among active users at one year compared to baseline regarding the questionnaires and clinical outcome variables. A higher proportion of girls' families were found among the active users (P< .001). A similar proportion of active users 7/68 (10%) had HbA1c >8 as in the comparison group 56/419 (13%) (paediatric .535)."

18-i) Subgroup analysis of comparing only users

"Active user analysis
In a separate analysis before and after the first year of access, active users were defined as those where someone in the family logged in five times or more during their first year with access to the portal. This cut-off level for active use was defined retrospectively taking into account the distribution of frequency of use.
The group of active users were compared to those with zero to four site visits during the same time period. Thus we merged data for the intervention group at baseline and after one year only (study year one), and for the previous control group before and after one year of access respectively (study year two)."

19) CONSORT: All important harms or unintended effects in each group

"The intervention group compared to the control group
No differences were found at baseline and after study year one between the intervention and control group, adolescents and parents respectively, regarding the outcome variables (HRQOL, empowerment, perception of quality of care regarding information measured by DISABKIDS, SWE-DES-SF-10 and QPP respectively, HbA1c, severe hypoglycaemia, frequency of blood glucose self-control). No differences were found at baseline and after study year one and two respectively, neither in the intervention group nor in the control group, for adolescents and parents respectively, regarding the same outcome variables. "

19-i) Include privacy breaches, technical problems

"However, there is a lack of logged data from August 30, 2008 due to problems with the data server."

19-ii) Include qualitative feedback from participants or observations from staff/researchers

"The portal had been developed through a user-centred design process which included iterative sessions with groups of patients and parents as well as with the involved diabetes teams [39, 40]. A prototype was piloted in 2005 and the portal Diabit was launched in April 2006."

DISCUSSION

20) CONSORT: Trial limitations, addressing sources of potential bias, imprecision, multiplicity of analyses

20-i) Typical limitations in ehealth trials

..."frequencies of use were limited by a password requirement having the function of a gatekeeper [38].
A limitation of the study is that the effect on patient and parent knowledge of diabetes was not evaluated."

21) CONSORT: Generalisability (external validity, applicability) of the trial findings

21-i) Generalizability to other populations

"the logged user behaviours and our qualitative evaluations indicate that a fully implemented Web 2.0 system including a larger population for a community and without passwords might be of great complementary value for both patients and professionals [38]. Future research also involving larger sample sizes and with multicentre collaboration might add knowledge on development of various effective educational interventions [17]."

21-ii) Discuss if there were elements in the RCT that would be different in a routine application setting

"the logged user behaviours and our qualitative evaluations indicate that a fully implemented Web 2.0 system including a larger population for a community and without passwords might be of great complementary value for both patients and professionals [38]. Future research also involving larger sample sizes and with multicentre collaboration might add knowledge on development of various effective educational interventions [17]."

22) CONSORT: Interpretation consistent with results, balancing benefits and harms, and considering other relevant evidence

22-i) Restate study questions and summarize the answers suggested by the data, starting with primary outcomes and process outcomes (use)

"The portal attracted all groups of users with great individual variation in frequency of use, and as expected not all were users. In contrast to research on structured patient education including Web 2.0, this study did not evaluate a directed intervention programme. The portal was a complementary information and communication resource for self-directed use whenever needed. Based on known information needs, developed through patient, parent and health practitioners' interaction, the portal merely offered a practitioner-driven high-quality alternative for online information and communication. Thus we performed this experiment as close to a future real-world resource as possible, leaving it to patients and parents to make their own decisions on use of the portal. In contrast to numerous other online resources though, the patients' local multi-professional paediatric diabetes teams created and/or verified the information contents. "
"The active users' data seems somewhat promising although the overall usage rate was low. As expected a higher frequency of use was related to shorter diabetes experience. The frequency of use might also relate to factors such as personal interest and motivation, and/or perceived health status and satisfaction with the traditional care and education."

22-ii) Highlight unanswered new questions, suggest future research

"Future research also involving larger sample sizes and with multicentre collaboration might add knowledge on development of various effective educational interventions [17]. Patient engagement and social marketing of new tools warrant more attention."

Other information

23) CONSORT: Registration number and name of trial registry

Trial registration
Current Controlled Trials, www.controlled-trials.com
ISRCTN92107365

24) CONSORT: Where the full trial protocol can be accessed, if available

Fig 4.
Please contact the authors for more information

25) CONSORT: Sources of funding and other support (such as supply of drugs), role of funders

The Linkoping Diabit study was a practitioner-driven project supported by the Medical Research Council of Southeast Sweden (FORSS) and by ALF Grants, County Council of Östergötland and the Swedish Child Diabetes Foundation (Barndiabetesfonden)

X26-i) Comment on ethics committee approval

"Ethic statement
The study was approved by the Research Ethics Committee of the Faculty of Health Science at Linköping University, Sweden. The trial was registered as ISRCTN92107365, www.controlled-trials.com
Basic information to adolescents and parents about the study was given by posted letters. They were informed in the letter about confidentiality and the right to withdraw without explanation. All participants including next of kin were required to return a signed consent form. Informed consent was also given by each participant in electronic form prior to the first visit to the portal."

x26-ii) Outline informed consent procedures

"Basic information to adolescents and parents about the study was given by posted letters. They were informed in the letter about confidentiality and the right to withdraw without explanation. All participants including next of kin were required to return a signed consent form. Informed consent was also given by each participant in electronic form prior to the first visit to the portal."

X26-iii) Safety and security procedures

"Masking
All personnel were blinded to group assignment for the first study year. Care providers took part in development of the portal, as described above, and were informed about the study but had no information on assignment. They were instructed to discuss any clinical issue raised by the patient as usual, without trying to identify the group to which the patient belonged."

X27-i) State the relation of the study team towards the system being evaluated

The authors declare that there is no duality of interest associated with this manuscript.
